# Long non-coding RNA LINC-PINT as a novel prognostic biomarker in human cancer: a meta-analysis and machine learning

**DOI:** 10.1038/s41598-024-57836-y

**Published:** 2024-03-29

**Authors:** Jie Lin, Li Chen, Dan Zhang

**Affiliations:** 1 Department of Hepatobiliary and Pancreatic Surgery, Jilin University Second Hospital, Changchun, Jilin China; 2 Department of Hepatobiliary Disease, Fuzong Clinical Medical College of Fujian Medical University, Fuzhou, Fujian China

**Keywords:** Cancer, LINC-PINT, Long non-coding RNA, Prognosis biomarker, Cancer, Computational biology and bioinformatics

## Abstract

Long intergenic non-protein coding RNA, P53 induced transcript (LINC-PINT) exhibits different expression patterns in the majority of tumors, yet its relationship with cancer prognosis remains a subject of debate. This study aims to comprehensively investigate the prognostic significance of LINC-PINT in diverse human cancer. A systematic search was conducted in PubMed, Embase, Cochrane Library, and Web of Science databases to identify pertinent studies exploring the correlation between LINC-PINT expression and cancer patients. Moreover, bioinformatics analysis and in vitro validation were used to validate the results of the meta-analysis and to investigate the potential oncogenic mechanism of LINC-PINT. The meta-analysis encompassed 8 studies, involving 911 patients. The pooled analysis demonstrated a significant association between upregulation of LINC-PINT expression and better survival (*P* = 0.002) during the cancers. Meanwhile, its downregulation was correlated with advanced tumor staging (*P* = 0.04) and tumor differentiation (*P* = 0.03). Additionally, bioinformatics analysis showed that LINC-PINT expression was observed to be linked with Tumor Mutational Burden (TMB) and Microsatellite Instability (MSI) in tumors, the results of bioinformatics were verified by qRT-PCR. And functional enrichment analysis hinted at its involvement in tumorigenesis and tumor progression. Dysregulated LICN-PINT expression is associated with the clinical prognostic and pathological features of various cancers, exhibiting substantial potential as a novel prognostic biomarker.

## Introduction

According to the 2020 global cancer statistics^1^ published by the World Health Organization, there were an estimated 19.3 million new cancer cases and nearly 10 million cancer-related deaths worldwide. Projections indicate that by the year 2040, the global cancer burden will surge to 28.4 million cases, signifying a substantial 47% increase compared to the figures recorded in 2020. Cancer continues to stand as a predominant cause of mortality, presenting a significant impediment to enhancing life expectancy across all nations. Despite the array of treatment modalities, such as surgery, radiotherapy, chemotherapy, and combination therapies, the overall prognosis for individuals afflicted with cancer remains dishearteningly bleak^2^. A primary contributor to this scenario lies in the paucity of sensitive and specific biomarkers that could aid in early detection of tumors. Consequently, the majority of patients are diagnosed when their cancer has already progressed to an advanced stage^3^. Consequently, the quest for novel cancer biomarkers and therapeutic targets assumes paramount clinical significance.

Long non-coding RNAs (LncRNAs) constitute a specific subclass of transcripts, characterized by a length surpassing 200 bp, and they lack identifiable open reading frames or the ability to encode proteins. Operating in the RNA form, they serve as pivotal regulators of gene expression imprints, functioning at multiple levels encompassing splicing, transcription, and the genome. Ongoing research endeavors have brought to light the intricate involvement of LncRNAs in an array of physiological and pathological processes occurring within the human body^4^. Remarkably, in the context of cancer, the dysregulated expression of LncRNAs assumes a key role, vigorously fostering and perpetuating the intricate course of tumor development and progression^5^.

LINC-PINT, a transcriptional product widely expressed in the human body and induced by p53, has been extensively studied for its role in regulating tumor cell proliferation through the induction of cell apoptosis and DNA damage^6^. Accumulating evidence^7^ has highlighted the dysregulation of LINC-PINT in various cancer types. Its upregulation in non-small cell lung cancer (NSCLC)^8^ and bladder cancer (BC)^9^ has been shown to exert a significant inhibitory effect on the malignant behavior of tumors. Nevertheless, intriguingly, contradictory finding^10^ have also emerged in specific tumor contexts, where increased LINC-PINT expression has been associated with adverse disease-free survival (DFS) and overall survival (OS) for patients.

In summary, there was no definitive conclusion regarding the association between LINC-PINT and the prognosis and clinical characteristics of tumors. Some studies presented divergent views on this matter. To address this discrepancy and surmount the limitations arising from small sample sizes in individual investigations, as well as to further ascertain the prognostic significance of LINC-PINT, we systematically selected relevant literature and conducted a meta-analysis to comprehensively evaluate the prognostic impact of LINC-PINT in diverse cancer types.

## Materials and methods

### Literature search strategy

The Preferred Reporting Items for Systematic Reviews and Meta-Analyses (PRISMA) guidelines were employed to guide the conduct and reporting of this meta-analysis. J.L. and L.C. conducted an exhaustive literature search in PubMed, Web of Science, Embase, and Cochrane Library, encompassing data from the inception of these databases up until April 21, 2023. No language restrictions were imposed during the search process. The search strategy encompassed a combination of Medical Subject Headings (MeSH) terms and free-text terms. Specifically, the search strategy utilized was (“neoplasms” or “carcinoma” or “prognosis” or “diagnosis” or “survival”) and (“LINC PINT” or “LINC PINT gene” or “long intervening non-coding RNA, p53 induced transcript”).

### Eligibility criteria

The inclusion criteria for this study were defined as follows: (1) Eligible studies that utilized qRT-PCR or RNA-seq to assess the expression of LINC-PINT in tumor tissues obtained from cancer patients; (2) Studies that involved patients with a confirmed cancer diagnosis and provided a comprehensive description of the association between LINC-PINT expression and survival outcomes or clinicopathological parameters (CP); (3) Studies that reported hazard ratios (HR) and corresponding 95% CI for prognostic indicators or allowed for indirect estimation based on survival curves. The following criteria were established for exclusion: (1) Non-clinical research studies, commentaries, letters, expert opinions, reviews, and case reports; (2) Studies with insufficient data extraction or ineffective data manipulation that hindered their suitability for the meta-analysis; (3) Studies where prognostic or clinical pathology data were solely derived from bioinformatics analysis, without direct clinical validation or experimental confirmation. The titles and abstracts of the literatures were independently reviewed by D.Z. (all the literatures) and J.L. (half the literatures) and L.C. (half the literatures) based the above criteria. The discrepancies were discussed with the third author (D.Z.).

### Data extraction and quality assessment

In this study, two researchers (J.L. and L.C.) independently performed a literature search and screening process adhering to the aforementioned inclusion and exclusion criteria. They meticulously extracted crucial information from the selected studies, including the first author’s name, publication year, geographic region of the study, sample size, cancer type under investigation, the employed detection method (qRT-PCR or RNA-seq), outcome measures assessed, the duration of follow-up in months, as well as HR and their corresponding 95% CI pertaining to the prognostic indicators analyzed. J.L. and L.C. independently performed data extraction and conducted a rigorous assessment of the included studies’ quality. If there are any discrepancies, they sought resolution through consultation with a third evaluator. For the evaluation of study quality, the esteemed Newcastle–Ottawa Scale (NOS) was employed (https://www.ohri.ca//programs/clinical_epidemiology/oxford.asp). This meticulous assessment scale encompassed three critical domains: the selection of study groups, comparability between these groups, and the rigorousness of outcome measurements. The highest attainable total score on this scale was 9 points, and based on the scores obtained, the studies were categorized as either low quality (3–4 points), medium quality (6–7 points), or high quality (7–9 points).

### Differential expression and survival analysis of LINC-PINT

We collected expression matrices and clinical information for 33 different types of cancer from The Cancer Genome Atlas (TCGA) database (https://portal.gdc.cancer.gov/)^11^. Additionally, we investigated the association between LINC-PINT expression and tumor staging using the UALCAN database^12^. Integrating the expression and survival data for these 33 cancer types, we conducted univariate Cox regression analysis to explore the potential links between LINC-PINT expression levels and OS, disease-specific survival (DSS), DFS, and progression-free survival (PFS) across various cancers. Subsequently, we used the Kaplan–Meier (K-M) plotter method, with the median expression value of LINC-PINT as the cutoff, to visualize the correlation between high and low LINC-PINT expression and survival prognosis in the aforementioned four survival categories.

### TMB, MSI, and functional enrichment analysis of LINC-PINT

We retrieved the data on tumor mutational burden (TMB) and microsatellite instability (MSI) in the pan-cancer context. Subsequently, investigating the correlation between LINC-PINT expression and these tumor characteristics. From the Gene Expression Profiling Interactive Analysis (GEPIA) database (http://gepia.cancer-pku.cn/index.html)^13^, the top 100 genes associated with LINC-PINT was identified. We conducted functional enrichment analysis on these genes to elucidate the potential pathways in which LINC-PINT may be involved, and summarized articles pertaining to experimentally validated pathways.

### Validation of LINC-PINT expression in vitro

To enhance the credibility of the conclusions, following the completion of the meta-analysis and bioinformatics validation, we conducted qRT-PCR experiments to validate the expression of LINC-PINT. Taking colorectal cancer and breast cancer as examples, we initially downloaded GSE9348 and GSE45827 from the GEO database (https://www.ncbi.nlm.nih.gov/geo/) as validation datasets. Subsequently, we selected the human colorectal cell line (NCM460), colorectal cancer cell line (SW480), human breast cell line (MCF10A), and breast cancer cell line (MCF7) to investigate the expression of LINC-PINT in different cancers. The above cell lines were cultured at 37 °C with 5% CO_2_.

The PCR primers synthesis and other experimental consumables were procured from Sangon Biotech Co., Ltd., Shanghai, China. Total RNA extraction was performed using the Trizol method, followed by cDNA synthesis using the Quanshijin reagent kit (Beijing Quanshijin Biotechnology Co, Ltd, China). The PCR reaction followed a two-step protocol provided by Bio-Rad Company (USA), and data quantification was conducted using the accompanying software. Finally, data visualization was carried out using GraphPad Prism 9.5 software. The primer sequences were as follows: Forward primer (GAACGAGGCAAGGAGCTAAA) and Reverse primer (AGCAAGGCAGAGAAACTCCA).

### Data processing and statistical analysis

The statistical analysis was conducted using Review Manager 5.2 software. To estimate HR and 95% CI, K-M curves from each included study were digitized using Engauge Digitizer 11.3 software. The survival outcomes were then derived from the logHR and standard error values. Additionally, the association between LINC-PINT expression levels and tumor CP (age, sex, differentiation grade, tumor size, TNM stage, and metastasis) was evaluated using odd ratio (OR) and their corresponding 95% CIs. Heterogeneity among the eligible studies was assessed through Q and I^2^ tests. Based on the observed heterogeneity among the studies, the appropriate effect model was selected. When no significant heterogeneity was detected (I^2^ < 50%, *P* > 0.1), a fixed-effect model was employed for the meta-analysis. Conversely, in the presence of significant heterogeneity (I^2^ ≥ 50%, *P* ≤ 0.1), a random-effects model was utilized. Begg’s and Egger’s tests, along with sensitivity analysis, were performed using Stata SE12.0 software. Begg’s and Egger’s tests were used to explore potential publication bias. In case of the presence of publication bias, the trim-and-fill method was applied to further assess the stability of the combined results. Additionally, sensitivity analysis was carried out to investigate the sources of heterogeneity and the robustness of the outcomes. In the meta-analysis, the high and low expression of LINC-PINT was primarily defined either directly in the articles or, for those articles that did not define expression thresholds, expression values were divided using OriginLab software (https://www.originlab.com/). Data extraction and analysis of LINC-PINT correlations were accomplished using Perl programming language (version 5.30.0) and R language (version 4.1.2). Differential analysis of target genes was conducted using the ggpubr, plyr, and ggsci packages. Subsequently, survival analysis of target genes was performed using the survival, forestplot, and survminer packages. For visualizing the role of LINC-PNT in cancer, fmsb, reshape2, and RColorBrewer were employed. Furthermore, functional enrichment analysis of target genes was executed using the limma, org.Hs.eg.db, clusterProfiler, and enrichplot packages. Throughout all statistical analyses, results with *P* < 0.05 were considered statistically significant. Levels of significance were indicated as *P* < 0.05, < 0.01, and < 0.001, which were denoted as "*", "**", and "***", respectively.

### Ethical statement

This study was registered in PROSPERO (registration number CRD42023449535).

## Results

### Literature selection

**Figure 1:**
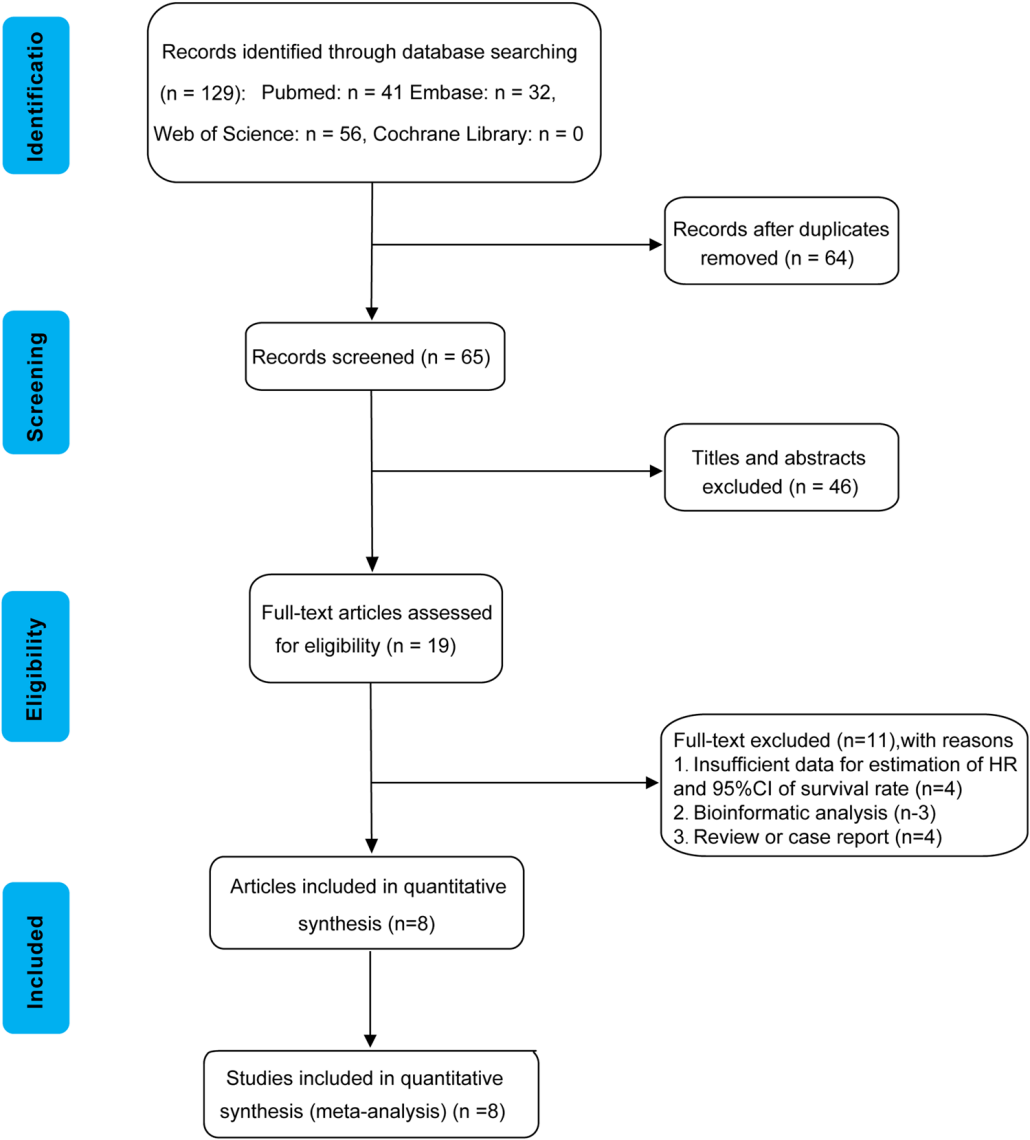
Flow chart of literature screening for this meta-analysis.

We conducted an extensive search across four databases, yielding a total of 129 studies (Pubmed = 41, Embase = 32, Web of Science = 56, Cochrane library = 0) that were initially identified. Following the removal of 64 duplicate articles, we scrutinized the titles and abstracts of the remaining studies, leading to the exclusion of 46 studies that were found to be irrelevant to our study. Subsequently, a thorough examination of the full texts of 19 articles was performed. Among these 19 articles, four were excluded from the analysis as they did not provide the necessary data to calculate HR and corresponding 95% CI. Additionally, three articles were excluded due to their primary focus on bioinformatics analysis, while four other articles were excluded as they were categorized either as review papers or case reports. Consequently, a total of eight articles^9,10,14–19^ were deemed eligible for inclusion in the meta-analysis. A flow chart depicting the literature screening process is shown in Fig. 1.

### Study characteristics and quality assessment

Table 1 presented a comprehensive overview of the characteristics of the studies included in our analysis. A total of 911 patients were enrolled across the diverse spectrum of cancer types, comprising pancreatic cancer, gastric cancer, renal cell carcinoma, esophageal cancer, bladder cancer, laryngeal squmaous cell carcinoma, and NSCLC. The primary focus of all the included studies was to investigate the correlation between LINC-PINT expression and OS, with follow-up periods spanning from 36 to 60 months. With only two exceptions, the vast majority of the studies also reported essential CP. Notably, the assessment of LINC-PINT expression across all studies was carried out through the application of qRT-PCR, a widely utilized quantitative method. Moreover, the evaluation of research quality, as measured by the NOS, indicated that all included studies achieved NOS scores of ≥ 6, signifying a collective standard of moderate to high quality. A visual representation of the research quality for the included literature can be found in Fig. S1.

**Table 1:** Characteristics of eligible studies in this meta-analysis.

Study	Year	Region	Tumor type	Sample size (low/high)	HR availability	Outcomes	Method	Follow-up months	NOS score
Li et al.^15^	2016	China	Pancreatic cancer	61 (27/34)	K–M curve	OS, CP	qRT-PCR	60	7
Feng et al.^14^	2019	China	Gastric cancer	72 (39/33)	K–M curve	OS	qRT-PCR	60	6
Duan et al.^10^	2019	China	Renal cell carcinoma	98 (16/82)	K–M curve	OS, PFS, CP	qRT-PCR	60	7
Lei et al.^16^	2019	China	Gastric cancer	78 (40/38)	K–M curve	OS	qRT-PCR	60	6
Zhang et al.^18^	2019	China	Esophageal cancer	337 (270/67)	K–M curve	OS, CP	qRT-PCR	60	6
Han et al.^9^	2021	China	Bladder Cancer	113 (56/57)	K–M curve	OS, CP	qRT-PCR	60	7
Yang et al.^17^	2021	China	Laryngeal squamous cell carcinoma	30 (15/15)	K–M curve	OS, CP	qRT-PCR	36	7
Zhang et al.^19^	2021	China	Non-small cell lung cancer	122 (64/58)	K–M curve	OS, CP	qRT-PCR	60	7

*OS* Overall survival, *PFS* Progression free survival, *CP* Clinicopathological parameters, *NOS* Newcastle–Ottawa scale, *K–M curve* Kaplan–Meier curve, *qRT-PCR* Quantitative real time polymerase chain reaction.

### Meta-analysis

#### Association between LINC-PINT and prognosis

**Figure 2:**
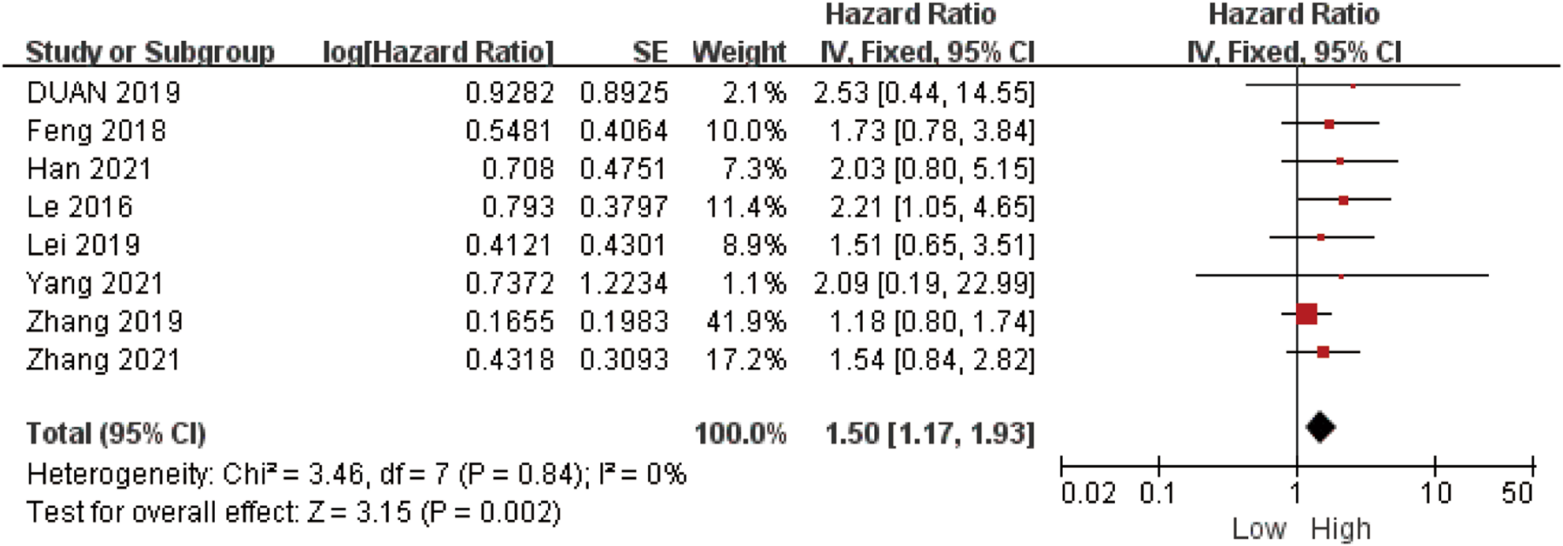
Forest plot demonstrating the relationships between LINC-PINT expression and OS.

In the analysis of 8 studies pertaining to OS, it was found that there was no significant statistical heterogeneity observed among these studies (*P* = 0.84, I^2^ = 0%). Therefore, a fixed-effects model was employed for the statistical analysis. The findings from this analysis demonstrated a notable and meaningful association between elevated LINC-PINT expression and improved OS [HR = 1.50, 95% CI (1.17, 1.93), *P* = 0.002, Fig. 2]. To gain deeper insights into the relationship between LINC-PINT expression levels and OS, we conducted comprehensive subgroup analyses based on the following factors: follow-up time (≥ 5 years or < 5 years), cancer system (digestive system or other), sample size of patients (≥ 80 cases or < 80 cases), and treatment modality (surgery or combination therapy). Remarkably, the results obtained from these subgroup analyses consistently supported the robust predictive value of LINC-PINT for OS in cancer patients, as it remained unaltered and consistent across the subgroups (Table 2, Fig. S2).

**Table 2:** Subgroup analysis of LINC-PINT expression an OS in cancer patients.

Subgroup analysis	No. of studies	No. of patients	Pooled HR (96% CI)	*P*	Hetarogeneity		Model
					I^2^ (%)	*P* value	
OS	8	911	1.50 (1.17,1.93)	0.002	0	0.84	Fixed
Follow-up time							
≥ 5 years	7	881	1.49 (1.16, 1.92)	0.002	0	0.76	Fixed
< 5 years	1	30	2.09 (0.19, 22.99)	0.55	0	0	Fixed
Tumor type							
Digestive system cancer	5	611	1.46 (1.10, 1.94)	0.008	0	0.56	Fixed
Others	3	300	1.65 (0.94, 2.87)	0.08	0	0.85	Fixed
Number of patients							
≥ 80	4	670	1.37 (1.01, 1.85)	0.04	0	0.60	Fixed
< 80	4	241	1.83 (1.17, 2.87)	0.008	0	0.92	Fixed
Therapy							
Surgery	7	839	1.48 (1.13, 1.92)	0.004	0	0.77	Fixed
Combined	1	72	1.73 (0.78, 3.84)	0.18	0	0	Fixed

#### Correlation of LINC-PINT expression with CP

**Figure 3:**
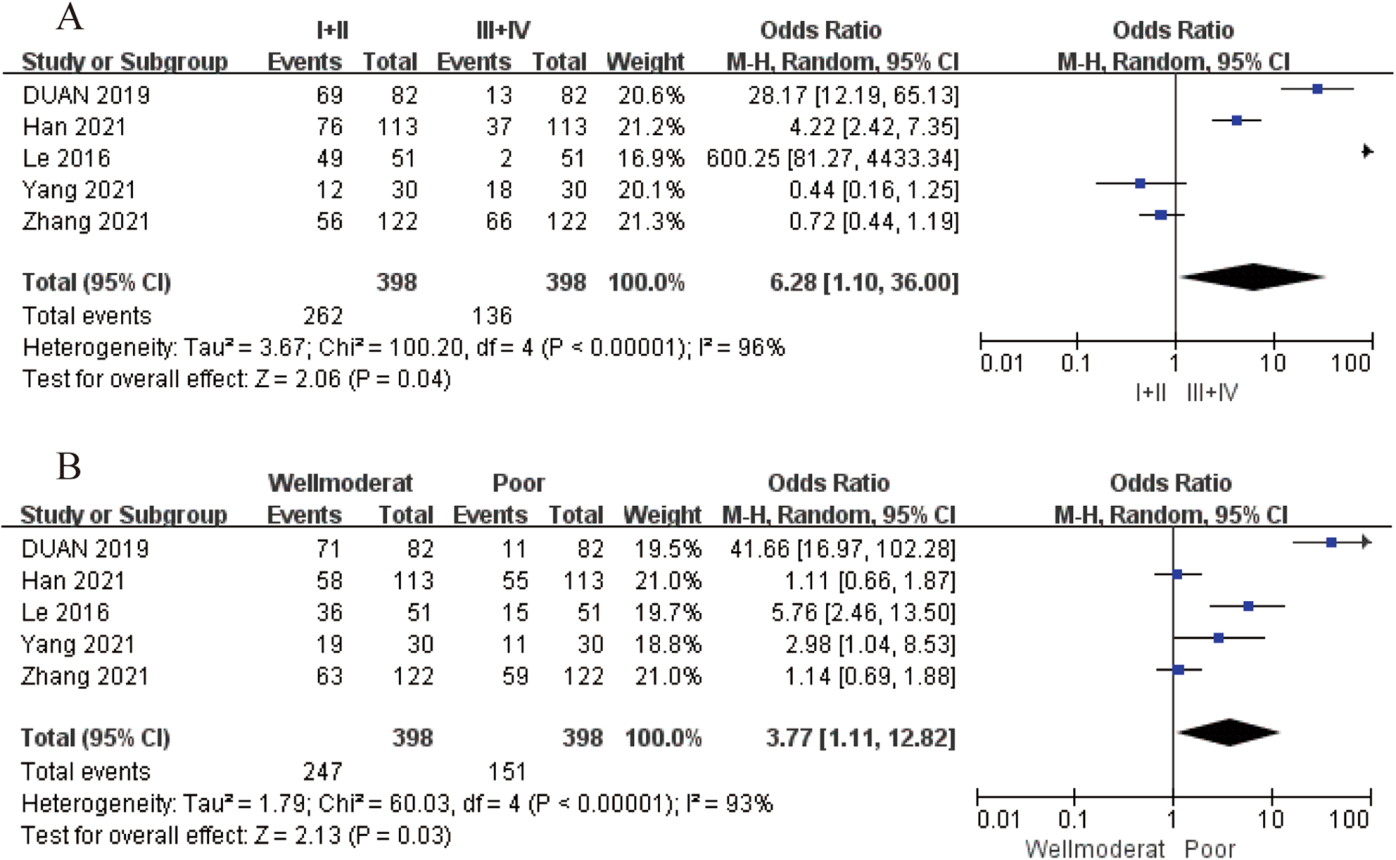
Forest plot of the relationship between low LINC-PINT expression and CP parameters. (**A**) Tumor stage, (**B**) tumor differentiation grade.

Among the 8 studies included in this analysis, 6 of them^9,10,15,17–19^ provided valuable data on the CP associated with low LINC-PINT expression, as comprehensively summarized in Table S1. Specifically, 5 of these studies^9,10,15,17,19^ confirmed that low LINC-PINT expression was remarkably linked to advanced tumor stage [OR = 6.28, 95% CI (1.10, 36.00), *P* = 0.04, Fig. 3A] and tumor differentiation grade [OR = 3.77, 95% CI (1.11, 12.82), *P* = 0.03, Fig. 3B]. However, no statistically significant associations were identified between low LINC-PINT expression and other crucial clinical factors such as age [OR = 1.39, 95% CI (0.89, 2.18), *P* = 0.15, Fig. S3A], gender [OR = 1.51, 95% CI (0.66, 3.49), *P* = 0.33, Fig. S3B], tumor size [OR = 0.65, 95% CI (0.14, 3.01), *P* = 0.58, Fig. S3C], and tumor metastasis [OR = 0.31, 95% CI (0.05, 2.02), *P* = 0.22, Fig. S3D].

Subsequently, we further examined the relationship between LINC-PINT expression and tumor staging by utilizing the UNCLAN database (Fig. S4). The results demonstrated that in certain specific tumor types, such as bladder urothelial carcinoma, breast invasive carcinoma, and lung squamous cell carcinoma, as the tumor stage progressed, there was a notable decrease in LINC-PINT expression. This finding is consistent with our previous research, further reinforcing the potential significance of LINC-PINT as a prognostic indicator in these particular cancer types.

#### Sensitivity analysis and publication bias

**Figure 4:**
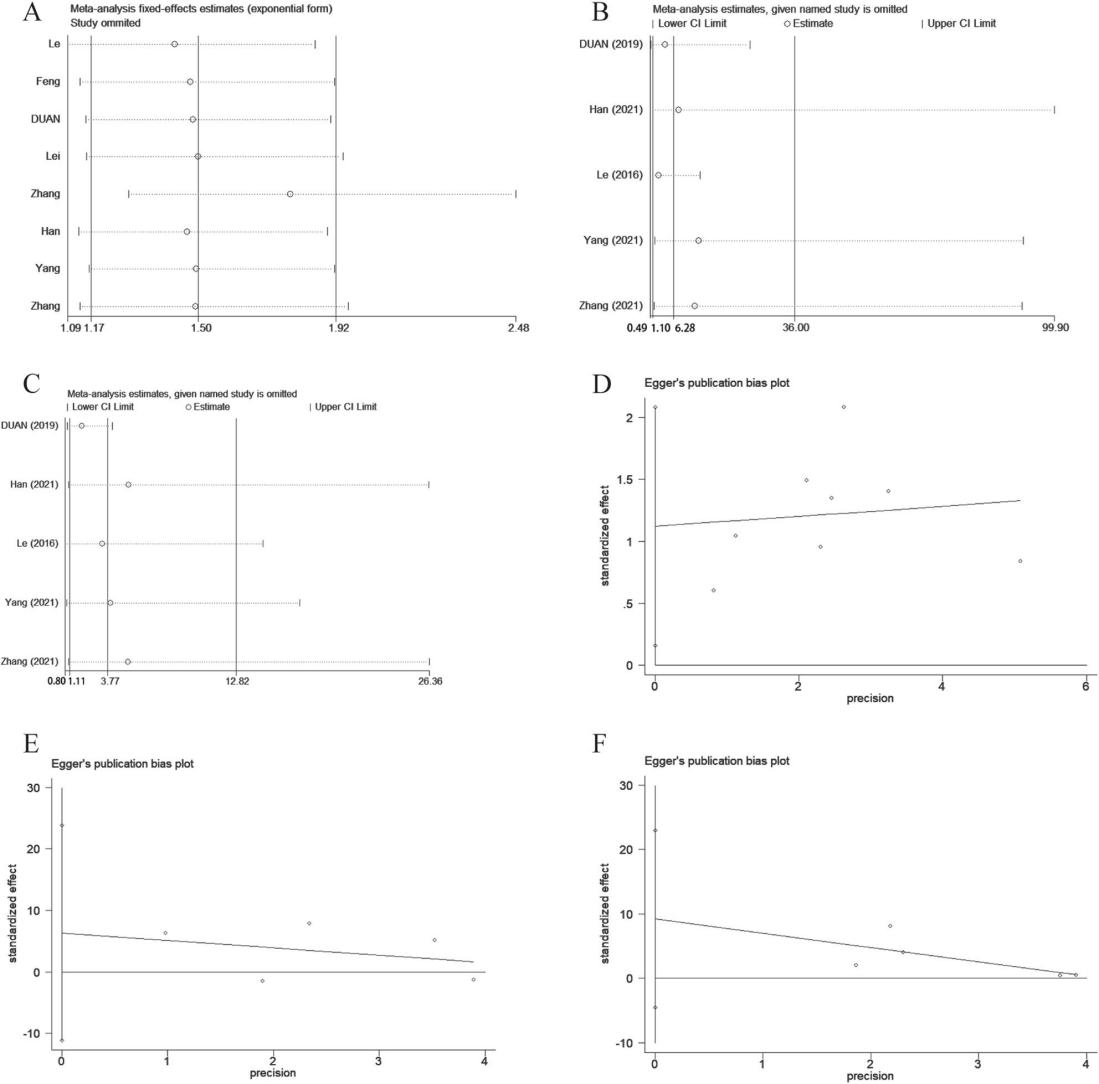
Sensitivity analysis of (**A**) OS, (**B**) tumor stage, (**C**) tumor differentiation grade. Egger’s publication bias plots of (**D**) OS, (**E**) tumor stage, (**F**) tumor differentiation grade.

To assess the robustness of our results, we conducted sensitivity analyses (Fig. 4, S5) by systematically excluding one or multiple studies at a time. Notably, these analyses demonstrated that the exclusion of any specific study did not significantly impact the overall results, thereby attesting to the stability and consistency of our conclusions. Furthermore, the application of Egger’s test revealed the presence of publication bias in the assessment of OS (Table S2, Fig. 4D). To further assess the reliability of the studies, we employed the trim-and-fill method. Specifically, for OS, the analysis indicated a potential absence of 4 studies, and upon adjusting for this potential publication bias, the resulting HR was calculated to be 1.35 [95% CI (1.076, 1.695), *P* = 0.01]. This important finding underscores the association of LINC-PINT upregulation with improved OS, thereby consolidating the credibility and trustworthiness of our research outcomes.

### Bioinformatics-analysis

#### Expression differences and prognostic value of LINC-PINT in human cancer

**Figure 5:**
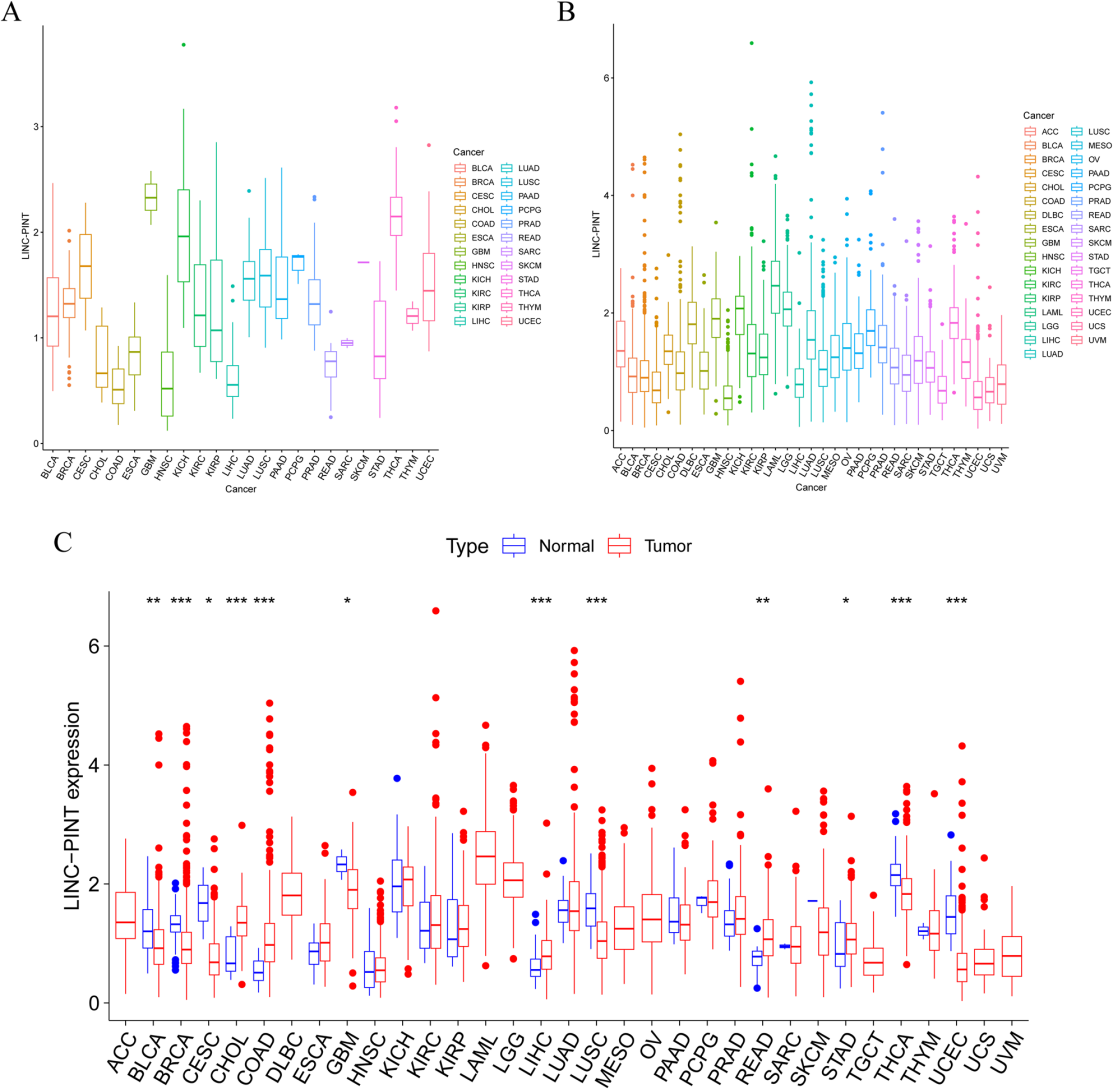
(**A**) Expression of LINC-PINT in normal tissues, (**B**) Expression of LINC-PINT in Tumor Tissues, (**C**) Differential expression of LINC-PINT in Pan-Cancer.

We initiated our investigation by scrutinizing the expression patterns of LINC-PINT within the extensive TCGA dataset, encompassing both normal tissues (Fig. 5A) and tumor tissues (Fig. 5B). Then, we analyzed differential expression of LINC-PINT between tumor and adjacent non-tumor tissues within the TCGA cohort (Fig. 5C). Our examination revealed a consistent downregulation of LINC-PINT across seven distinct tumor types, namely Bladder Urothelial Carcinoma (BLCA), Breast Invasive Carcinoma (BRCA), Cervical squamous cell carcinoma and endocervical adenocarcinoma (CESC), Glioblastoma Multiforme (GBM), Lung Squamous Cell Carcinoma (LUSC), Thyroid Carcinoma (THCA), and Uterine Corpus Endometrial Carcinoma (UCEC). Remarkably, in stark contrast, we observed a notable upregulation of LINC-PINT in Cholangiocarcinoma (CHOL), Colon Adenocarcinoma (COAD), Liver Hepatocellular Carcinoma (LIHC), Rectum Adenocarcinoma (READ), and Stomach Adenocarcinoma (STAD).

**Figure 6:**
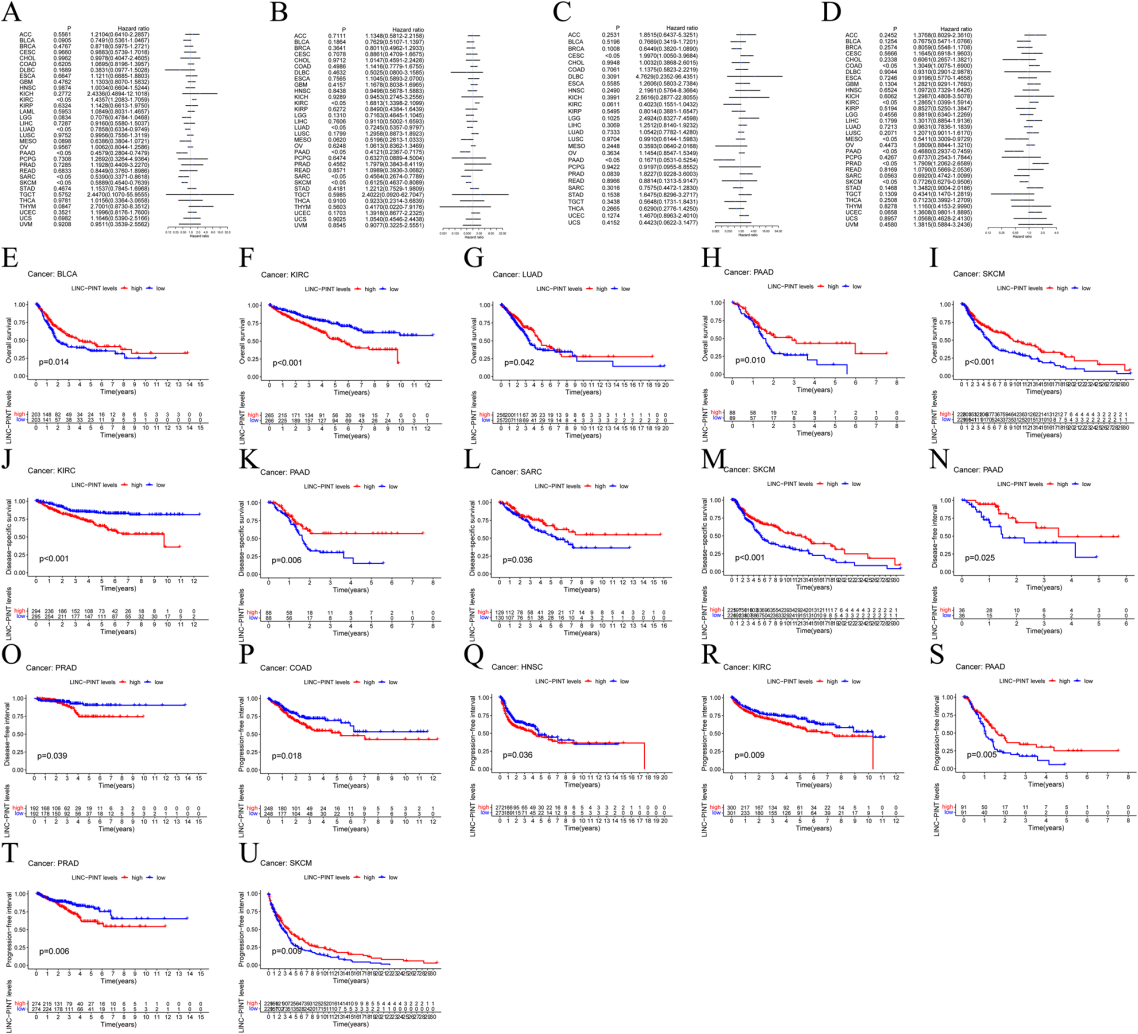
COX method is used to analyze the relationship between the expression of LINC-PINT and various survival times in pan-cancer. (**A**) OS, (**B**) DSS, (**C**) DFI, (**D**) PFI. The K-M plotter method was used to draw the relationship between the high and low expression of LINC-PINT in pan-cancer and the survival time. (**E**) OS of BLCA, (**F**) OS of KIRC, (**G**) OS of LUAD, (**H**) OS of PAAD, (**I**) OS of SKCM, (**J**) DSS of KIRC, (**K**) DSS of PAAD, (**L**) DSS of SARC, (**M**) DSS of SKCM, (**N**) DFI of PAAD, (**O**) DFI of PRAD, (**P**) PFI of COAD, (**Q**) PFI of HNSC, (**R**) PFI of KIRC, (**S**) PFI of PAAD, (**T**) PFI of PRAD, (**U**) PFI of SKCM.

In order to further elucidate the prognostic value of LINC-PINT across various cancers, we conducted a univariate COX regression analysis to explore the potential correlation between LINC-PINT expression levels and tumor survival outcomes. Our investigation unveiled a myriad of roles that LINC-PINT assumes in influencing OS within diverse cancer types. Specifically, in Kidney Renal Clear Cell Carcinoma (KIRC), LINC-PINT emerged as a deleterious gene (HR > 1, *P* < 0.05), significantly exerting an adverse impact on OS (Fig. 6A). Conversely, in Lung Adenocarcinoma (LUAD), Pancreatic Adenocarcinoma (PAAD), Sarcoma (SARC), and Skin Cutaneous Melanoma (SKCM), LINC-PINT assumed the role of a favorable gene (HR < 1, *P* < 0.05), thereby demonstrating an encouraging association with improved OS outcomes (Fig. 6A). Moreover, in the context of DSS, LINC-PINT was identified as a detrimental gene (HR > 1, *P* < 0.05) in KIRC (Fig. 6B). However, a converse trend was observed in LUAD, PAAD, SARC, and SKCM (HR < 1, *P* < 0.05), thus indicative of a positive impact on DSS (Fig. 6B). Furthermore, when scrutinizing the Disease-Free Interval (DFI), LINC-PINT emerged as an unfavorable prognostic factor (HR > 1, *P* < 0.05) in CESC (Fig. 6C). Conversely, in the context of PAAD, LINC-PINT assumed a favorable role (HR < 1, *P* < 0.05), thereby holding promise as a predictor for better DFI outcomes (Fig. 6C). Finally, as for Progression-Free Interval (PFI) (Fig. 6D), LINC-PINT exhibited a beneficial effect (HR < 1, *P* < 0.05) in Mesothelioma (MESO), PAAD, and SKCM, thus offering potential therapeutic implications in these malignancies. Conversely, in COAD, KIRC and Prostate Adenocarcinoma (PRAD), LINC-PINT acted as a detrimental factor (HR > 1, *P* < 0.05).

In our pursuit to comprehensively explore the impact of LINC-PINT expression on tumor survival outcomes, we conducted additional K-M analyses (Fig. 6E–U). Across the majority of cancer investigated, heightened expression of LINC-PINT was consistently correlated with extended OS periods, a compelling observation indicative of its potential beneficial role in these contexts. However, in KIRC, high LINC-PINT expression was significantly associated with poorer OS (*P* < 0.001), DSS (*P* < 0.001), and PFI (*P* = 0.009). Similarly, in PRAD, elevated LINC-PINT expression was linked to unfavorable outcomes in terms of DFI (*P* = 0.039) and PFI (*P* = 0.006). Likewise, in COAD, augmented LINC-PINT expression was associated with adverse PFI (*P* = 0.018). Moreover, in Head and Neck Squamous Cell Carcinoma (HNSC), heightened LINC-PINT expression was associated with poorer PFI (*P* = 0.036). These results indicate that even the same gene may play different roles in different cancers.

#### Functional enrichment analysis, TMB, and MSI analysis of LINC-PINT

We utilized the GEPIA tool to obtain the top 100 genes exhibiting the strongest correlation with LINC-PINT (Table S3). Subsequently, we subjected these 100 genes to Gene Ontology (GO) and Kyoto Encyclopedia of Genes and Genomes (KEGG) enrichment analysis. The GO analysis results revealed that LINC-PINT-associated genes are enriched in several pathways and processes related to RNA regulation, such as mRNA processing, RNA splicing, and regulation of mRNA metabolic process (Fig. S6A). Furthermore, the KEGG analysis indicated that LINC-PINT-associated genes are intriguingly associated with the PPAR signaling pathway and Th17 cell differentiation (Fig. S6B).

TMB, quantifying the total number of mutations per megabase of DNA, has recently emerged as a promising biomarker for predicting the effectiveness of immunotherapy in cancer treatment^20^. Our research findings (Fig. S6C) revealed a positive correlation between LINC-PINT expression and TMB in several cancer types, including Adrenocortical Carcinoma (ACC), Thymoma (THYM), SKCM, and Esophageal Carcinoma (ESCA) (correlation coefficient > 0, *P* < 0.05). Conversely, in UCEC, THCA, Testicular Germ Cell Tumors (TGCT), STAD, KIRC, COAD, and BRCA, LINC-PINT expression exhibited a negative correlation with TMB (correlation coefficient < 0, *P* < 0.05). MSI refers to the spontaneous gain or loss of nucleotides in tumor cells^21^. Studies have suggested that MSI can serve as a predictive marker for the sensitivity of cancers to specific chemotherapy drugs^22^. As illustrated in Fig. S6D, LINC-PINT expression demonstrated a positive correlation with MSI in THCA, SKCM, PRAD, LUSC, LUAD, Lower Grade Glioma (LGG), HNSC, and BRCA (correlation coefficient > 0, *P* < 0.05). Conversely, in TGCT, STAD, and COAD, MSI displayed a negative correlation with LINC-PINT expression (correlation coefficient < 0, *P* < 0.05).

**Figure 7:**
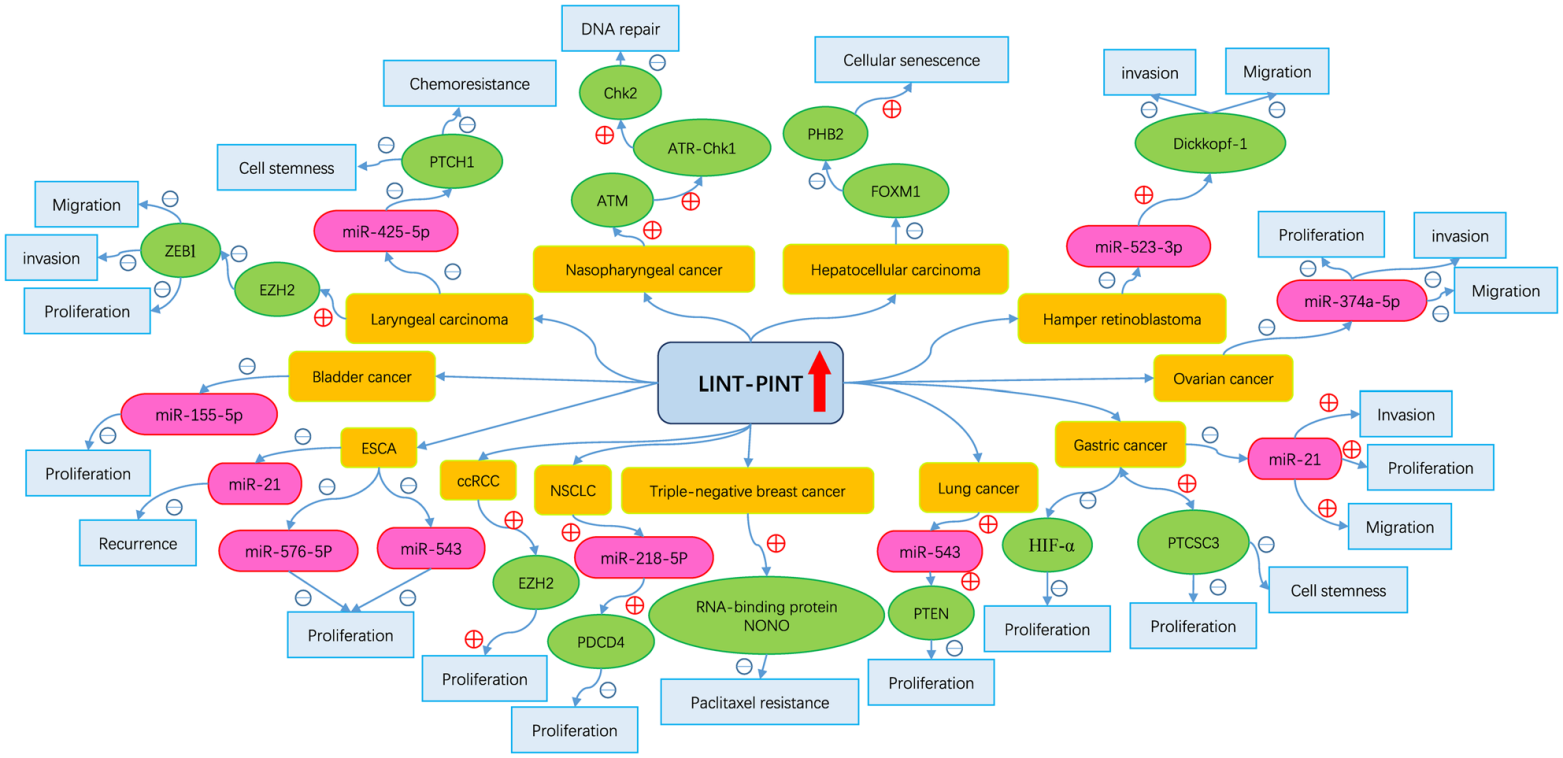
Schematic representation of the regulatory mechanism of LINC-PINT in pan-cancer.

While the exploration of potential pathways in which LINC-PINT may be involved has been conducted as described above, the precise regulatory mechanisms through which LINC-PINT participates in tumor modulation remain elusive. We have compiled a summarized representation of the potential pathways in which LINC-PINT may be implicated (Fig. 7, Table S4). As depicted in the figure, elevated levels of LINC-PINT have been associated with the changes of downstream miRNAs, leading to the deceleration of tumor proliferation and invasion in certain cancers, such as lung cancer and bladder cancer^8,9,23^. Moreover, specific pathways have been found to be influenced by changes in LINC-PINT expression. For example, in gastric cancer, upregulated LINC-PINT can inhibit the HIF-α pathway, thereby impeding cancer cell proliferation^24^. Furthermore, LINC-PINT has been shown to cooperate with other LncRNAs in the joint regulation of tumor progression^16^. Collectively, these findings underscore the significant role of LINC-PINT in the physiological and pathological processes of cancer.

#### Additional validation sets and in vitro experiments

**Figure 8:**
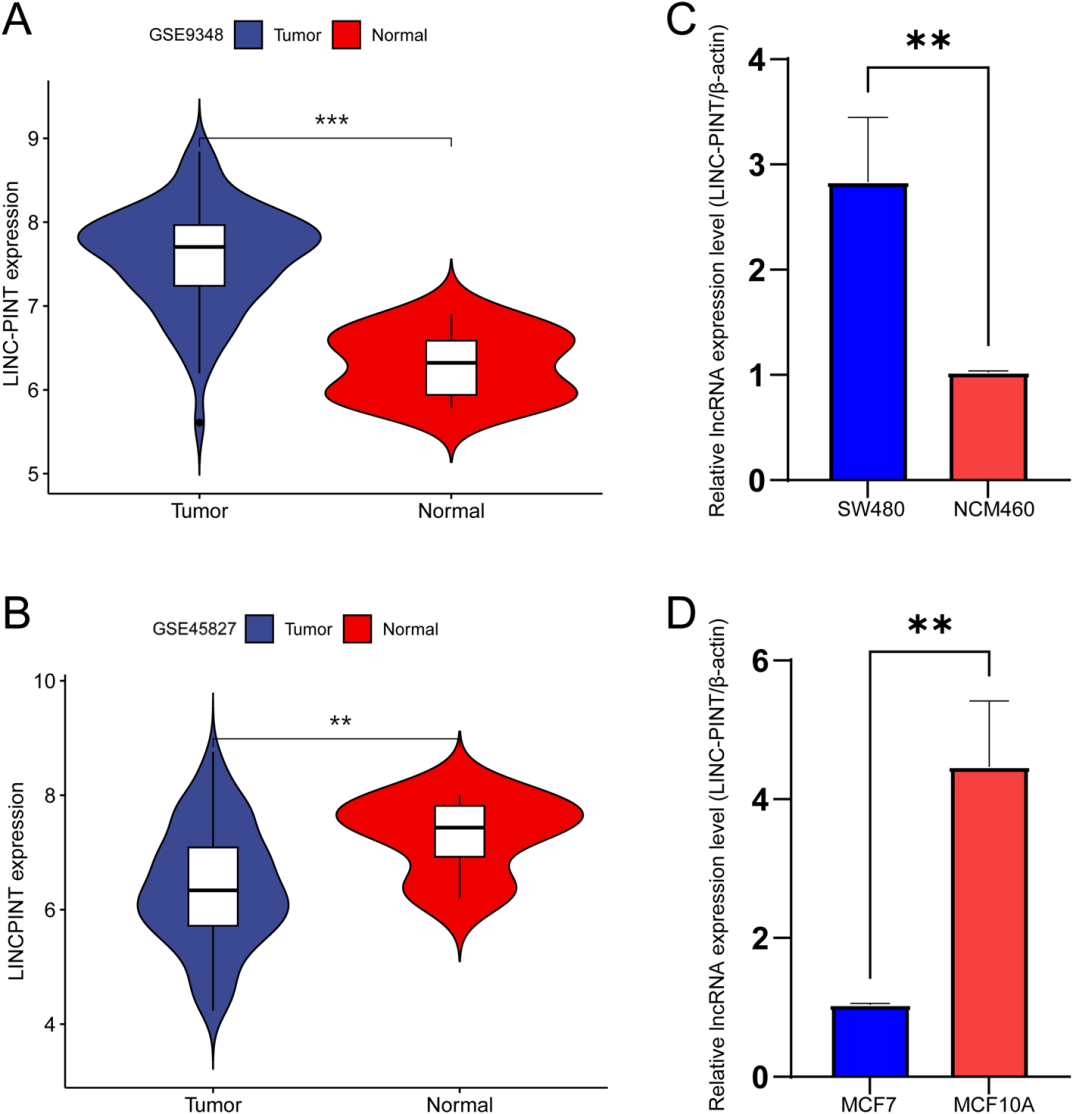
(**A**) The expression of LINC-PINT in the GSE9348 dataset, (**B**) The expression of LINC-PINT in the GSE45827 database, (**C**) qRT-PCR Results of LINC-PINT in colorectal cancer cell lines, (**D**) qRT-PCR results of LINC-PINT in breast cancer cell lines.

To further bolster the reliability of our conclusions, exemplified by colorectal cancer and breast cancer, our GEO validation results indicated high expression of LINC-PINT in our colorectal cancer cells and low expression in breast cancer cells (Fig. 8A,B), consistent with our TCGA findings. Additionally, qRT-PCR results revealed expression differences of LINC-PINT between tumor cell lines and normal cell lines (Fig. 8C,D), aligning with our bioinformatics analysis findings.

## Discussion

With the continuous advancement of medical technology and the distinctive tumor-specific characteristics of LncRNAs, these molecules have gradually emerged as effective biomarkers for the diagnosis and treatment of cancer^25^. A growing number of evidence demonstrates the close association between dysregulated LncRNA expression and a multitude of tumor features, including proliferation, apoptosis, invasion, and metastasis^26–28^. Through various intricate mechanisms, LncRNAs actively contribute to the process of tumorigenesis, encompassing the regulation of downstream microRNAs^29,30^, DNA methylation modifications^31^, and the induction of tumor drug resistance^32^. Given the indispensable role of LncRNAs in tumor development, the systematic identification of robustly tumor-associated LncRNAs holds tremendous potential in advancing precision oncology.

Some existing studies have demonstrated the close correlation between LINC-PINT dysregulation and tumor progression and treatment effect. To comprehensively summarize these relationships, we have classified them into four distinct categories as follows: Firstly, LINC-PINT actively participates in the regulation of tumor proliferation, invasion, and metastasis by the following ways. (1). It functions as a miRNA sponge, thereby intricately modulating tumor invasion^8,9,23,33–35^. (2). It exerts its influence on tumor proliferation through specific molecular pathways, such as the HIF-a pathway^24^. (3). It also engages in collaborative interactions with other LncRNAs, collectively fostering tumor proliferation^16^. Secondly, LINC-PINT plays a crucial role in the regulation of tumor cell stemness by engaging in interplay with other LncRNAs^16^ and effectively governing downstream miRNAs and their associated molecules^35^. Thirdly, LINC-PINT exerts regulatory control over specific pathways, such as the ATM/ATR-Chk1/Chk2 pathway, significantly influencing tumor cell DNA repair mechanisms^36^. Lastly, LINC-PINT exerts discernible effects on tumor drug sensitivity. It orchestrates the expression of downstream miRNAs, which in turn promote the generation of specific proteins, thereby modulating the responsiveness of tumor cells to chemotherapy drugs^36^. Moreover, it regulates the activity of specific proteins, such as RNA-binding protein NONO, critically regulating the sensitivity of cancer cells to paclitaxel treatment^37^. However, it is noteworthy that certain investigations have reported that elevated LINC-PINT expression is associated with a better prognosis in cancer patients^8^. Thus, the prospect of LINC-PINT serving as a pan-cancer biomarker remains unknown. To shed light on this intriguing question, we have undertaken a comprehensive investigation that entails meta-analysis and bioinformatics analysis, aiming to elucidate the clinical significance and oncogenic mechanisms of LINC-PINT in the context of cancer.

In our meta-analysis, we observed a significant association between elevated LINC-PINT expression and improved OS in cancer patients. Bioinformatics analyses further demonstrated that elevated LINC-PINT expression correlates with longer predicted survival in certain cancers such as BLCA, LUAD, PAAD, SARC, and SKCM. Conversely, in distinct tumor categories such as KIRC, PRAD, COAD, and HNSC, heightened LINC-PINT expression was linked to poorer prognostic outcomes. These intriguing findings collectively indicate that LINC-PINT may hold promising potential as a prognostic biomarker for cancer patients, with its predictive value being partly contingent on the particular cancer type under consideration. Furthermore, our meta-analysis indicated that low LINC-PINT expression is positively correlated with advanced TNM staging in tumors. Subsequent validation in our database confirmed these meta-analysis results, demonstrating that LINC-PINT downregulation is associated with tumor progression in specific cancers, such as prostate cancer, breast cancer, and lung cancer. Meanwhile, given the crucial impact of TMB and MSI alterations on the efficacy and outcomes of immunotherapies, our research findings have revealed compelling connections between LINC-PINT alterations and TMB as well as MSI in select tumor types. These valuable insights collectively advocate the consideration of LINC-PINT as a promising therapeutic target for novel anticancer treatments.

While this study provides a comprehensive understanding of the clinical prognosis and oncogenic mechanisms of LINC-PINT in cancer, there are also several limitations that should be acknowledged. Firstly, inherent factors, such as diverse cancer types, variations in detection techniques, and variations in follow-up duration, may contribute to heterogeneity in the meta-analysis. To mitigate the impact of such heterogeneity, future investigations will adopt more stringent inclusion/exclusion criteria for literature studies and pay more attention to subgroup analyses. Although our results were limited by sample size, they still provided preliminary insights into the potential role of LINC-PINT in the prognosis of cancer patients. This study could still offer valuable preliminary information for future research. We recommended that future studies in this field conduct more large-scale, high-quality research to further validate our findings and enhance credibility. Secondly, the calculation of HR and corresponding 95%CI based on survival curves might exhibit reduced precision due to the unavailability of direct HR values in some studies. Consequently, data had to be extracted using the graphical method from KM curves, possibly introducing inherent bias. Thirdly, this study predominantly focused on examining LINC-PINT expression in tumor tissues, leaving the potential of LINC-PINT as a non-invasive diagnostic or prognostic biomarker in circulation unclear. Moreover, despite the novel findings stemming from bioinformatics analyses, further in vitro and in vivo studies are warranted to validate the molecular mechanisms by which LINC-PINT modulates tumor development and drug resistance.

## Conclusion

To sum up, our study highlights LINC-PINT as a tumor key gene, and its expression is closely associated with malignant features and clinical outcomes in cancer patients. Additionally, LINC-PINT may exert its oncogenic role through various mechanisms, including immune regulation and miRNA modulation. Overall, these findings indicate that LINC-PINT holds not only the potential to serve as a reliable clinical biomarker for cancer diagnosis and prognosis but also as a promising target for precision therapeutic interventions.

### Supplementary Information

Supplementary Tables.

Supplementary Figures.

## Data Availability

The datasets generated during the current study are available in The Cancer Genome Atlas (TCGA) database (https://portal.gdc.cancer.gov/) and the Gene Expression Profiling Interactive Analysis (GEPIA) database (http://gepia.cancer-pku.cn/index.html).

## Abbreviations

LINC-PINT: Long intergenic non-protein coding RNA, P53 induced transcript
HR: Hazard ratios
OR: Odds ratios
CI: Confidence interval
TMB: Tumor mutational burden
MSI: Microsatellite instability
LncRNAs: Long non-coding RNAs
NSCLC: Non-small cell lung cancer
BC: Bladder cancer
DFS: Disease-free survival
OS: Overall survival
CP: Clinicopathological parameters
NOS: Newcastle–Ottawa scale
TCGA: The Cancer Genome Atlas
DSS: Disease-specific survival
PFS: Progression-free survival
K–M: Kaplan–Meier
BLCA: Bladder urothelial carcinoma
BRCA: Breast invasive carcinoma
CESC: Cervical squamous cell carcinoma and endocervical adenocarcinoma
GBM: Glioblastoma multiforme
LUSC: Lung squamous cell carcinoma
THCA: Thyroid carcinoma
UCEC: Uterine corpus endometrial carcinoma
CHOL: Cholangiocarcinoma
COAD: Colon adenocarcinoma
LIHC: Liver hepatocellular carcinoma
READ: Rectum adenocarcinoma
STAD: Stomach adenocarcinoma
KIRC: Kidney renal clear cell carcinoma
LUAD: Lung adenocarcinoma
PAAD: Pancreatic adenocarcinoma
SARC: Sarcoma
SKCM: Skin cutaneous melanoma
DFI: Disease-free interval
PFI: Progression-free interval
PRAD: Prostate adenocarcinoma
HNSC: Head and neck squamous cell carcinoma
ACC: Adrenocortical carcinoma
THYM: Thymoma
ESCA: Esophageal carcinoma
TGCT: Testicular germ cell tumors
LGG: Lower grade glioma
