# Supplementary material for: Long non-coding RNA LINC-PINT as a novel prognostic biomarker in human cancer: a meta-analysis and machine learning
Source: Sci Rep. 2024 Mar 29;14:7483. doi: 10.1038/s41598-024-57836-y (PMC10980720; doi:10.1038/s41598-024-57836-y)
Supplement: Supplementary file 1 — Supplementary Tables. [file 41598_2024_57836_MOESM1_ESM.docx]

Table S1.The correlation between LINC-PINT expression and clinicopathologic parameters features.

Table S2.Results of Begg’s and Egger’s tests for publication bias.

If P＜0.05, the results are in bold.

Table S3. The Top 100 Genes Most Closely Associated with LINC-PINT

| Gene Symbol | Gene ID | PCC |
| --- | --- | --- |
| RP5-1057I20.4 | ENSG00000268069.2 | 0.73 |
| ARMCX4 | ENSG00000196440.11 | 0.71 |
| MAN2C1 | ENSG00000140400.14 | 0.7 |
| SLC25A27 | ENSG00000153291.15 | 0.7 |
| WDR19 | ENSG00000157796.17 | 0.7 |
| EZH1 | ENSG00000108799.12 | 0.69 |
| POU6F1 | ENSG00000184271.15 | 0.69 |
| ZNF540 | ENSG00000171817.16 | 0.68 |
| RP11-7F17.8 | ENSG00000273729.1 | 0.68 |
| PNISR | ENSG00000132424.14 | 0.68 |
| RP11-87H9.4 | ENSG00000280077.1 | 0.67 |
| CLK1 | ENSG00000013441.15 | 0.67 |
| KLHDC1 | ENSG00000197776.7 | 0.67 |
| RP13-516M14.10 | ENSG00000275888.1 | 0.67 |
| RRN3P1 | ENSG00000248124.7 | 0.66 |
| ZNF767P | ENSG00000133624.13 | 0.66 |
| SYNE1 | ENSG00000131018.22 | 0.66 |
| LENG8 | ENSG00000167615.16 | 0.66 |
| RUFY3 | ENSG00000018189.12 | 0.66 |
| TTC14 | ENSG00000163728.10 | 0.66 |
| ARGLU1 | ENSG00000134884.13 | 0.65 |
| NICN1 | ENSG00000145029.11 | 0.65 |
| SAFB2 | ENSG00000130254.11 | 0.65 |
| APBB3 | ENSG00000113108.17 | 0.65 |
| AC005519.4 | ENSG00000258559.2 | 0.65 |
| LINC00894 | ENSG00000235703.5 | 0.65 |
| ZNF471 | ENSG00000196263.7 | 0.65 |
| RP5-855D21.3 | ENSG00000272812.1 | 0.65 |
| ZNF76 | ENSG00000065029.14 | 0.65 |
| GPRASP1 | ENSG00000198932.12 | 0.65 |
| RP11-106M3.2 | ENSG00000260729.1 | 0.64 |
| PHF1 | ENSG00000112511.17 | 0.64 |
| CELF6 | ENSG00000140488.14 | 0.64 |
| LINC00893 | ENSG00000241769.7 | 0.64 |
| TUBGCP6 | ENSG00000128159.11 | 0.64 |
| RXRB | ENSG00000204231.10 | 0.64 |
| ZMAT1 | ENSG00000166432.14 | 0.63 |
| CTD-2270P14.1 | ENSG00000260565.6 | 0.63 |
| ZNF83 | ENSG00000167766.18 | 0.63 |
| PKMP3 | ENSG00000220563.1 | 0.63 |
| RBM6 | ENSG00000004534.14 | 0.63 |
| LUC7L | ENSG00000007392.16 | 0.63 |
| DDX17 | ENSG00000100201.18 | 0.63 |
| NPHP3 | ENSG00000113971.18 | 0.63 |
| CLCN6 | ENSG00000011021.21 | 0.62 |
| TNS2 | ENSG00000111077.17 | 0.62 |
| AHSA2 | ENSG00000173209.22 | 0.62 |
| RP11-972P1.7 | ENSG00000280328.1 | 0.62 |
| TSPYL2 | ENSG00000184205.14 | 0.62 |
| ARHGEF17 | ENSG00000110237.3 | 0.62 |
| RBM5 | ENSG00000003756.16 | 0.62 |
| SH3BP5-AS1 | ENSG00000224660.1 | 0.62 |
| RP11-64K12.8 | ENSG00000259211.1 | 0.62 |
| ZSCAN18 | ENSG00000121413.12 | 0.62 |
| KIAA0895L | ENSG00000196123.12 | 0.61 |
| TBRG1 | ENSG00000154144.12 | 0.61 |
| RP11-228B15.4 | ENSG00000225032.5 | 0.61 |
| RIC3 | ENSG00000166405.14 | 0.61 |
| RP11-44F14.6 | ENSG00000279722.1 | 0.61 |
| RP5-855D21.1 | ENSG00000272240.1 | 0.61 |
| RANP4 | ENSG00000225125.2 | 0.61 |
| SRSF5 | ENSG00000100650.15 | 0.61 |
| RP11-441O15.3 | ENSG00000224934.2 | 0.61 |
| AC005154.7 | ENSG00000244480.1 | 0.61 |
| CHD2 | ENSG00000173575.18 | 0.61 |
| TAF1C | ENSG00000103168.16 | 0.61 |
| DMTF1 | ENSG00000135164.18 | 0.6 |
| RP11-59C5.3 | ENSG00000273599.1 | 0.6 |
| RP11-311P8.2 | ENSG00000232186.1 | 0.6 |
| SIN3B | ENSG00000127511.9 | 0.6 |
| TRIM52 | ENSG00000183718.5 | 0.6 |
| KLF3-AS1 | ENSG00000231160.9 | 0.6 |
| LINC00115 | ENSG00000225880.5 | 0.6 |
| RP11-1114A5.4 | ENSG00000232611.1 | 0.6 |
| RAD51-AS1 | ENSG00000245849.6 | 0.6 |
| PAN2 | ENSG00000135473.14 | 0.6 |
| AC114730.11 | ENSG00000235351.1 | 0.6 |
| U2AF1L4 | ENSG00000161265.14 | 0.6 |
| ING5 | ENSG00000168395.14 | 0.6 |
| MLLT6 | ENSG00000275023.4 | 0.6 |
| AKAP8L | ENSG00000011243.17 | 0.6 |
| WDR6 | ENSG00000178252.17 | 0.6 |
| RBMS3 | ENSG00000144642.20 | 0.59 |
| MAGI2-AS3 | ENSG00000234456.7 | 0.59 |
| MSANTD2 | ENSG00000120458.9 | 0.59 |
| MICU3 | ENSG00000155970.11 | 0.59 |
| RNPC3 | ENSG00000185946.15 | 0.59 |
| CROCCP3 | ENSG00000080947.14 | 0.59 |
| RP4-800G7.2 | ENSG00000244560.6 | 0.59 |
| RP1-59D14.5 | ENSG00000263345.1 | 0.59 |
| CREBZF | ENSG00000137504.13 | 0.59 |
| FAM160B2 | ENSG00000158863.21 | 0.59 |
| RP3-406P24.5 | ENSG00000279926.1 | 0.59 |
| RP3-329A5.8 | ENSG00000272374.1 | 0.59 |
| ITPR1-AS1 | ENSG00000231249.1 | 0.59 |
| TTLL3 | ENSG00000214021.15 | 0.59 |
| AC005104.3 | ENSG00000223374.1 | 0.59 |
| EXD3 | ENSG00000187609.15 | 0.59 |
| AC005253.2 | ENSG00000268030.1 | 0.59 |
| RP11-346C20.3 | ENSG00000271009.2 | 0.59 |

Table S4:Molecular mechanism of LINC-PINT in various tumors

| Cancers | Regulatory relationship | Phenotype | Reference |
| --- | --- | --- | --- |
| Nasopharyngeal cancer | ATM/ATR-Chk1/Chk2 | DNA repair | ^1^ |
| Hepatocellular carcinoma | FOXM1/PHB2 | cellular senescence | ^2^ |
| Hamper retinoblastoma | miR-523-3p/Dickkopf-1 | invasion and migration | ^3^ |
| Ovarian cancer | miR-374a-5p | proliferation, migration and invasion | ^4^ |
| Gastric cancer | miR-21 | proliferation, migration, and invasion | ^5^ |
|  | PTCSC3 | tumor growth and cancer cell stemness | ^6^ |
|  | HIF‑1α | proliferation | ^7^ |
| Lung cancer | miR-543/PTEN | proliferation | ^8^ |
| Triple-negative breast cancer | RNA-binding protein NONO | paclitaxel resistance | ^9^ |
| NSCLC | miR-218-5p/PDCD4 | proliferation | ^10^ |
| ccRCC | EZH2 | cell proliferation | ^11^ |
| ESCA | miR-543 and miR-576-5p | proliferation | ^12^ |
|  | miRNA-21 | recurrence | ^13^ |
| Bladder Cancer | miR-155-5p | proliferation | ^14^ |
| Laryngeal carcinoma | miR-425-5p/PTCH1/SHH | cell stemness and chemoresistance | ^15^ |
|  | EZH2/ZEB1 | proliferation, migration and invasion | ^16^ |

1. Y. H. Wang *et al.*, LINC-PINT impedes DNA repair and enhances radiotherapeutic response by targeting DNA-PKcs in nasopharyngeal cancer. *Cell Death Dis* **12**, 454 (2021).

2. X. Xiang *et al.*, Cellular senescence in hepatocellular carcinoma induced by a long non-coding RNA-encoded peptide PINT87aa by blocking FOXM1-mediated PHB2. *Theranostics* **11**, 4929-4944 (2021).

3. X. Zhou *et al.*, LncRNA Linc-PINT inhibits miR-523-3p to hamper retinoblastoma progression by upregulating Dickkopf-1 (DKK1). *Biochem Biophys Res Commun* **530**, 47-53 (2020).

4. T. Hao, S. Huang and F. Han, LINC-PINT suppresses tumour cell proliferation, migration and invasion through targeting miR-374a-5p in ovarian cancer. *Cell Biochem Funct* **38**, 1089-1099 (2020).

5. H. Feng *et al.*, Long noncoding RNA LINC-PINT is inhibited in gastric cancer and predicts poor survival. *J Cell Biochem* **120**, 9594-9600 (2019).

6. L. Hong *et al.*, LncRNA PTCSC3 Inhibits Tumor Growth and Cancer Cell Stemness in Gastric Cancer by Interacting with lncRNA Linc-pint. *Cancer Manag Res* **11**, 10393-10399 (2019).

7. L. Hong *et al.*, Linc‑pint overexpression inhibits the growth of gastric tumors by downregulating HIF‑1α. *Mol Med Rep* **20**, 2875-2881 (2019).

8. S. Wang *et al.*, LINC-PINT alleviates lung cancer progression via sponging miR-543 and inducing PTEN. *Cancer Med* **9**, 1999-2009 (2020).

9. J. Chen *et al.*, Long non-coding RNA LINC-PINT attenuates paclitaxel resistance in triple-negative breast cancer cells via targeting the RNA-binding protein NONO. *Acta Biochim Biophys Sin (Shanghai)* **52**, 801-809 (2020).

10. L. Zhang *et al.*, Long noncoding RNA LINC-PINT inhibits non-small cell lung cancer progression through sponging miR-218-5p/PDCD4. *Artif Cells Nanomed Biotechnol* **47**, 1595-1602 (2019).

11. J. Duan *et al.*, Long noncoding RNA LINC-PINT promotes proliferation through EZH2 and predicts poor prognosis in clear cell renal cell carcinoma. *Onco Targets Ther* **12**, 4729-4740 (2019).

12. L. Zhang *et al.*, Linc-PINT acted as a tumor suppressor by sponging miR-543 and miR-576-5p in esophageal cancer. *J Cell Biochem* **120**, 19345-19357 (2019).

13. H. Rong *et al.*, Downregulation of lncRNA LINC-PINT Participates in the Recurrence of Esophageal Squamous Cell Carcinoma Possibly by Interacting miRNA-21. *Cancer Biother Radiopharm* **36**, 273-279 (2021).

14. X. Han, J. Liu, Y. Liu, L. Mou and C. Li, LINC-PINT Inhibited Malignant Progression of Bladder Cancer by Targeting miR-155-5p. *Cancer Manag Res* **13**, 4393-4401 (2021).

15. Z. Yuan *et al.*, Long noncoding RNA LINC-PINT regulates laryngeal carcinoma cell stemness and chemoresistance through miR-425-5p/PTCH1/SHH axis. *J Cell Physiol* **234**, 23111-23122 (2019).

16. X. Yang *et al.*, LncRNA LINC-PINT Inhibits Malignant Behaviors of Laryngeal Squamous Cell Carcinoma Cells via Inhibiting ZEB1. *Pathol Oncol Res* **27**, 584466 (2021).
