# Supplementary material for: Long non-coding RNA LINC-PINT as a novel prognostic biomarker in human cancer: a meta-analysis and machine learning
Source: Sci Rep. 2024 Mar 29;14:7483. doi: 10.1038/s41598-024-57836-y (PMC10980720; doi:10.1038/s41598-024-57836-y)
Supplement: Supplementary file 2 — Supplementary Figures. [file 41598_2024_57836_MOESM2_ESM.docx]

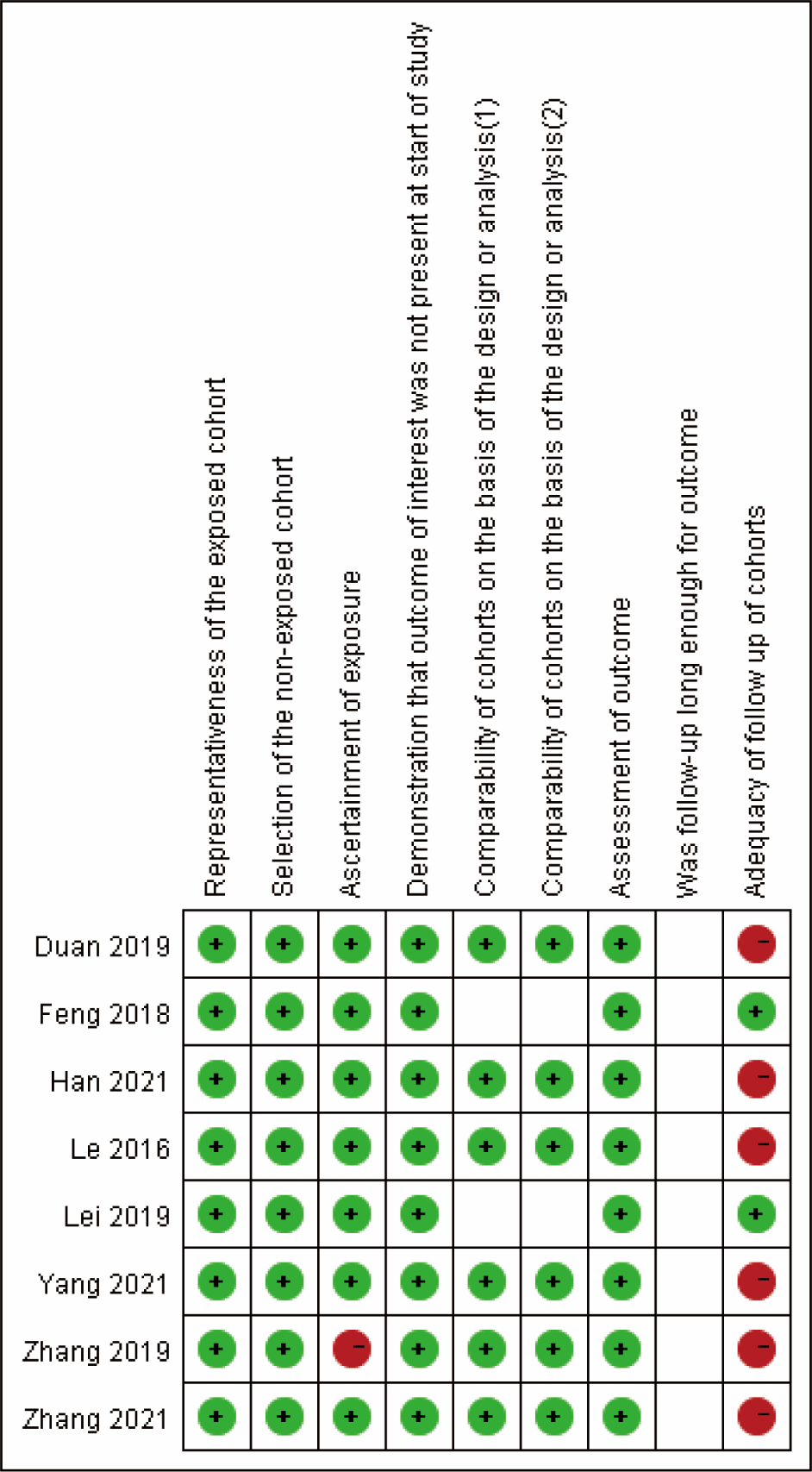


Figure S1. Result of quality assessment of included studies based on the NOS.


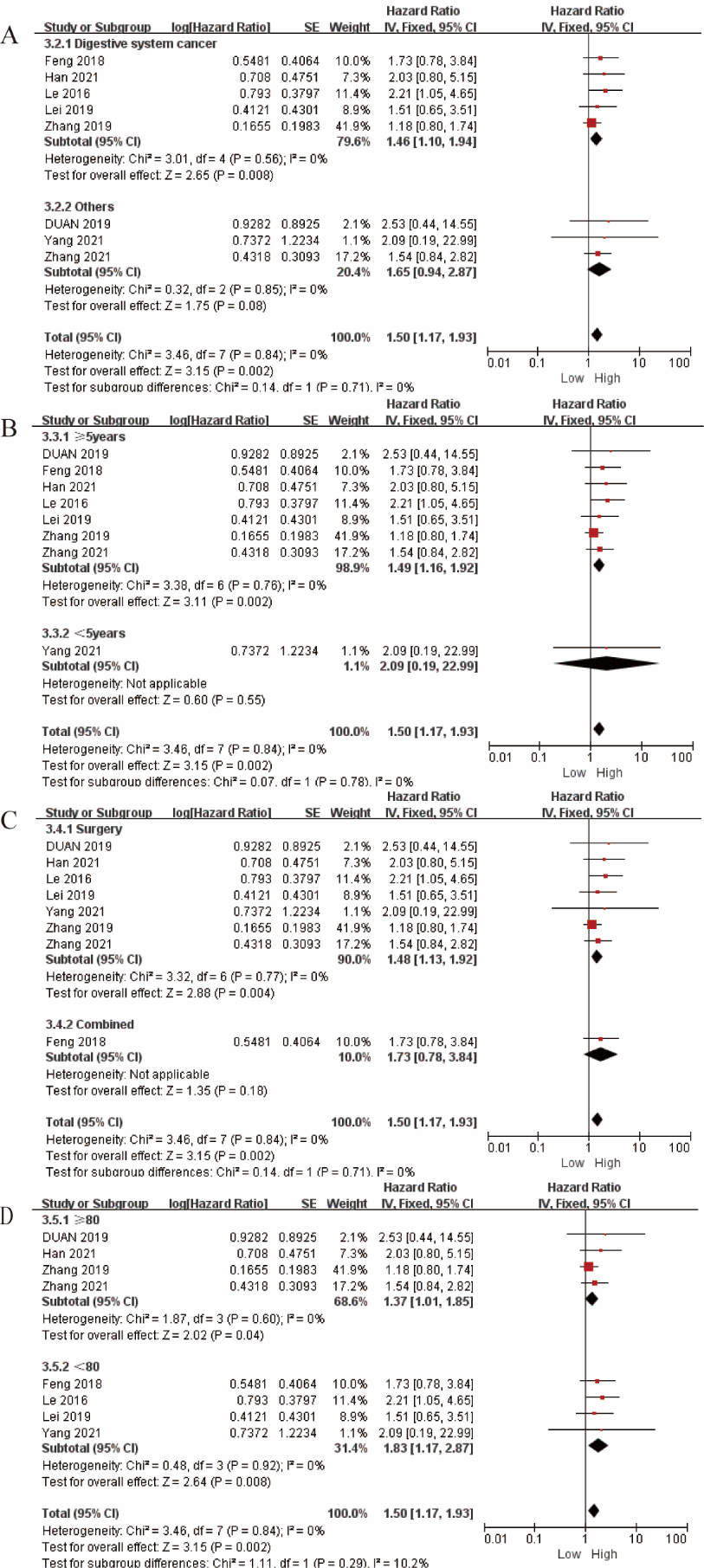


Figure S2. Forest plot demonstrating the relationships between LINC-PINT expression and (A) cancer system, (B) follow-up time, (C) treatment modality, (D) sample size of patients.


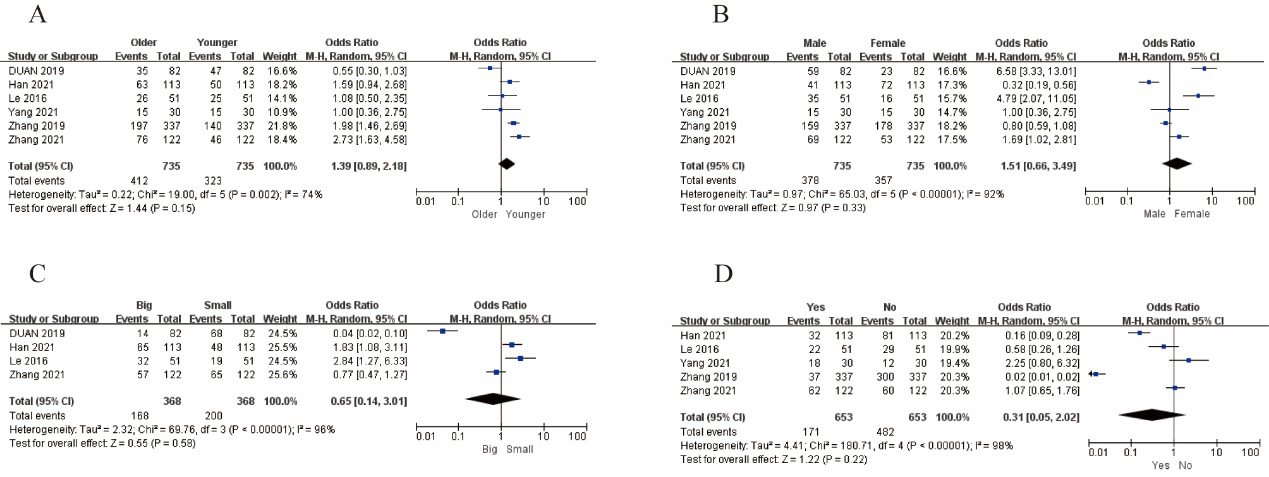


Figure S3. Forest plot of the relationship between low LINC-PINT expression and CP: (A) age, (B) gender, (C) tumor size, (D) tumor metastasis.


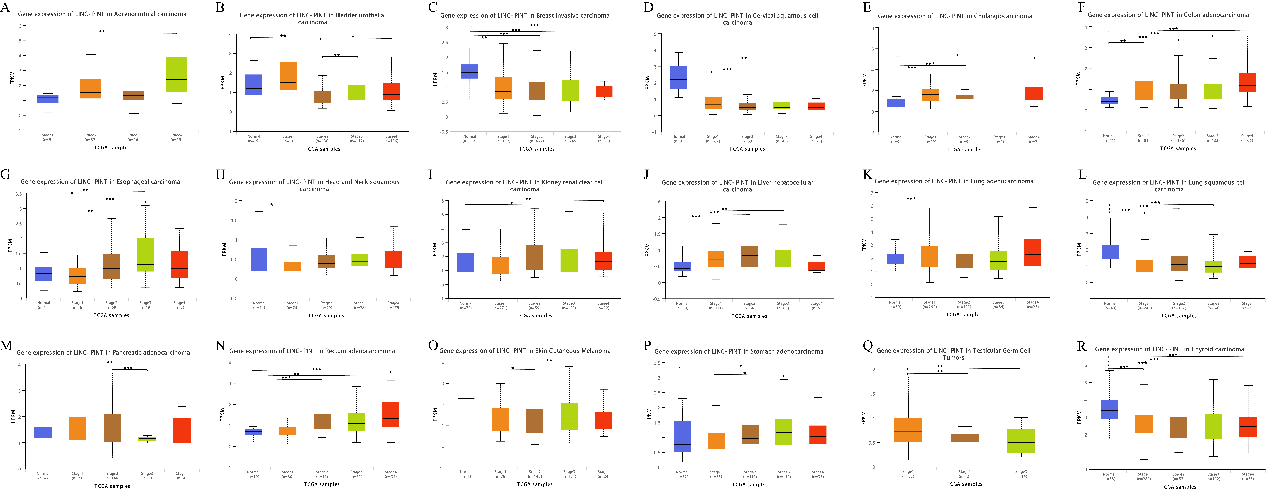


Figure S4. The relationship between the expression of LINC-PINT in cancer and its clinical features: (A) Adrenocortical carcinoma, (B) Bladder urothelial carcinoma, (C) Breast invasive carcinoma, (D) Cervical squamous cell carcinoma, (E) Cholangiocarcinoma, (F) Colon adenocarcinoma, (G) Esophageal carcinoma, (H) Head and Neck squamous carcinoma, (I) Kidney renal clear cell carcinoma, (J) Liver hepatocellular carcinoma, (K) Lung adenocarcinoma, (L) Lung squamous cell carcinoma, (M) Pancreatic adenocarcinoma, (N) Rectum adenocarcinoma, (O) Skin Cutaneous Melanoma, (P) Stomach adenocarcinoma, (Q) Testicular Germ Cell Tumors, (R) Thyroid carcinoma. P < 0.05, < 0.01, < 0.001 respectively mean "*", "* *" and "* * *".


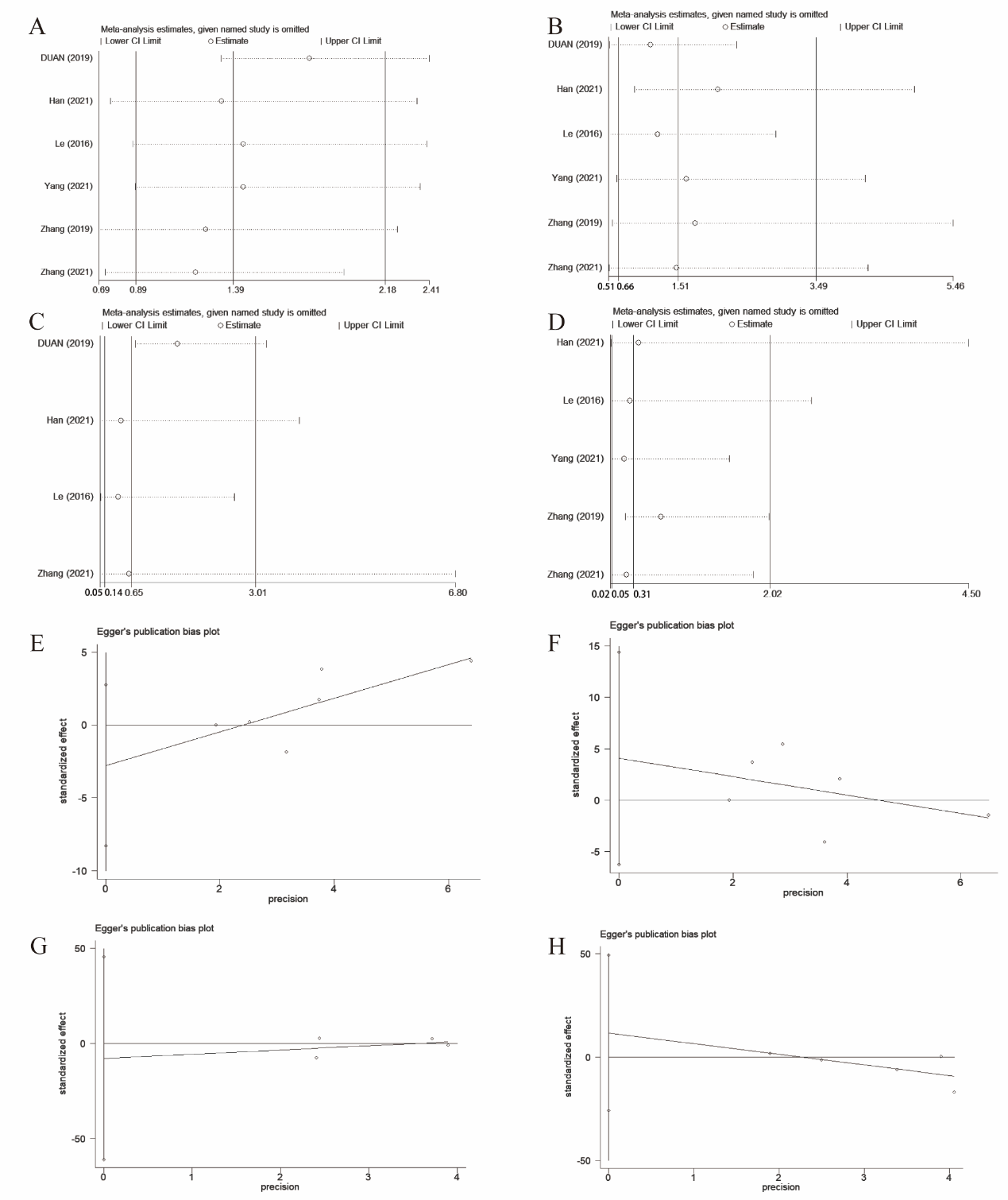


Figure S5. Sensitivity analysis of (A) age, (B) gender, (C) tumor size, (D) tumor metastasis. Egger’s publication bias plots of (E) age, (F) gender, (G) tumor size, (H) tumor metastasis.


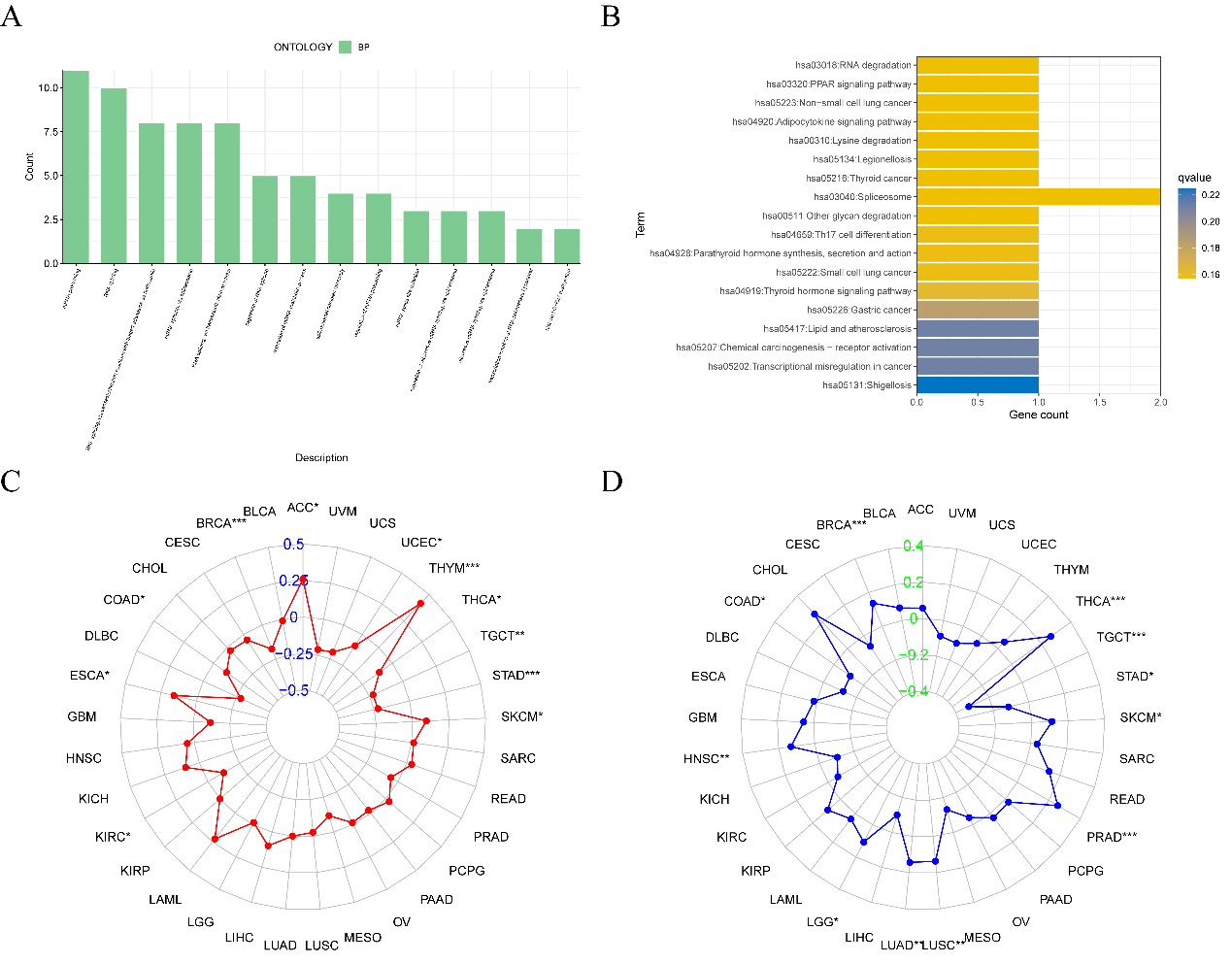


Figure S6 (A) GO analysis of LINC-PINT-associated genes, (B) KEGG analysis of LINC-PINT-associated genes, (C) The relationship between LINC-PINT expression and pan-cancer TMB, (D) The correlation between LINC-PINT expression and pan-cancer MSI.
